# Experimental study exploring the factors that promote rib fragility in the elderly

**DOI:** 10.1038/s41598-021-88800-9

**Published:** 2021-04-29

**Authors:** Christian Liebsch, Shamila Hübner, Marco Palanca, Luca Cristofolini, Hans-Joachim Wilke

**Affiliations:** 1 Institute of Orthopaedic Research and Biomechanics, Trauma Research Centre Ulm, University of Ulm, Helmholtzstraße 14, 89081 Ulm, Germany; 2 Department of Industrial Engineering, School of Engineering and Architecture, Alma Mater Studiorum - Università Di Bologna, Bologna, Italy

**Keywords:** Experimental models of disease, Medical research, Risk factors

## Abstract

Rib fractures represent a common injury type due to blunt chest trauma, affecting hospital stay and mortality especially in elderly patients. Factors promoting rib fragility, however, are little investigated. The purpose of this in vitro study was to explore potential determinants of human rib fragility in the elderly. 89 ribs from 13 human donors (55–99 years) were loaded in antero-posterior compression until fracture using a material testing machine, while surface strains were captured using a digital image correlation system. The effects of age, sex, bone mineral density, rib level and side, four global morphological factors (e.g. rib length), and seven rib cross-sectional morphological factors (e.g. cortical thickness, determined by μCT), on fracture load were statistically examined using Pearson correlation coefficients, Mann–Whitney U test as well as Kruskal–Wallis test with Dunn-Bonferroni post hoc correction. Fracture load showed significant dependencies (*p* < 0.05) from bone mineral density, age, antero-posterior rib length, cortical thickness, bone volume/tissue volume ratio, trabecular number, trabecular separation, and both cross-sectional area moments of inertia and was significantly higher at rib levels 7 and 8 compared to level 4 (*p* = 0.001/0.013), whereas side had no significant effect (*p* = 0.989). Cortical thickness exhibited the highest correlation with fracture load (r = 0.722), followed by the high correlation of fracture load with the area moment of inertia around the longitudinal rib cross-sectional axis (r = 0.687). High correlations with maximum external rib surface strain were detected for bone volume/tissue volume ratio (r = 0.631) and trabecular number (r = 0.648), which both also showed high correlations with the minimum internal rib surface strain (r =  − 0.644/ − 0.559). Together with rib level, the determinants cortical thickness, area moment of inertia around the longitudinal rib cross-sectional axis, as well as bone mineral density exhibited the largest effects on human rib fragility with regard to the fracture load. Sex, rib cage side, and global morphology, in contrast, did not affect rib fragility in this study. When checking elderly patients for rib fractures due to blunt chest trauma, patients with low bone mineral density and the mid-thoracic area should be carefully examined.

## Introduction

Clinical treatment of rib fractures has gained growing importance due to the rising number of falls in elderly people, causing about 75% of all traumatic injuries in patients aged over 60 years^1^. Previous investigations exhibited increased mortality following rib fractures in advanced age, accounting for more than 20% of patients aged over 65 years, but only for about 10% in younger patients^2–4^. While age and number of fractured ribs were found to be predictive factors for morbidity and mortality after blunt chest trauma^5^, about 20% of all patients suffer from chronic pain due to rib pseudarthrosis^6,7^.

Despite representing an apparent clinical issue, mechanisms and determinants promoting rib fractures have been insufficiently investigated. Recently published findings of an epidemiological study showed that old age, osteoporosis, prior fractures, and falls represent risk factors for rib fractures^8^. Moreover, several experimental and numerical studies investigated single factors affecting rib stability and fragility by focusing on few specific parameters such as age^9–13^, sex^10–13^, cortical thickness^13–15^, or rib geometry^13,16,17^, while Agnew et al.^13^ additionally analyzed rib level and bone mineral density as possible promoting factors. However, the effects of all relevant patient-specific and morphological factors on rib fragility in their entirety have been poorly studied so far. Knowledge about determinants for rib fragility could help to identify risk factors as part of clinical anamnesis and stationary treatment of elderly patients. Moreover, information about rib fragility is essential regarding future in vitro and in silico studies investigating rib fracture mechanisms, which could help to develop novel safety devices for elderly people and novel rib fracture treatment strategies.

The purpose of the present in vitro study therefore was to explore determinants of fragility of human ribs from elderly donors. The significance of the following factors was tested: age, sex, bone mineral density, rib level, rib cage side, global morphological factors, such as rib length and width, and local, cross-sectional morphological factors, such as the cortical and trabecular thickness.

## Results

### Fracture types and locations

After fracturing, the vast majority of the 89 investigated ribs exhibited transverse fractures as defined by Meinberg et al.^18^, while 54 of the 60 transverse fractures were infractions, i.e. incomplete fractures. Of the remaining 29 ribs, 25 showed oblique fractures (19 infractions), whereas only 4 ribs had multifragment fractures (1 infraction). Fracture locations were detected in the antero-lateral section as defined by Liebsch et al.^19^ in 88 of the 89 investigated ribs, while only one rib fractured in the posterior section.

### Effects of age, sex, and bone mineral density

**Figure 1:**
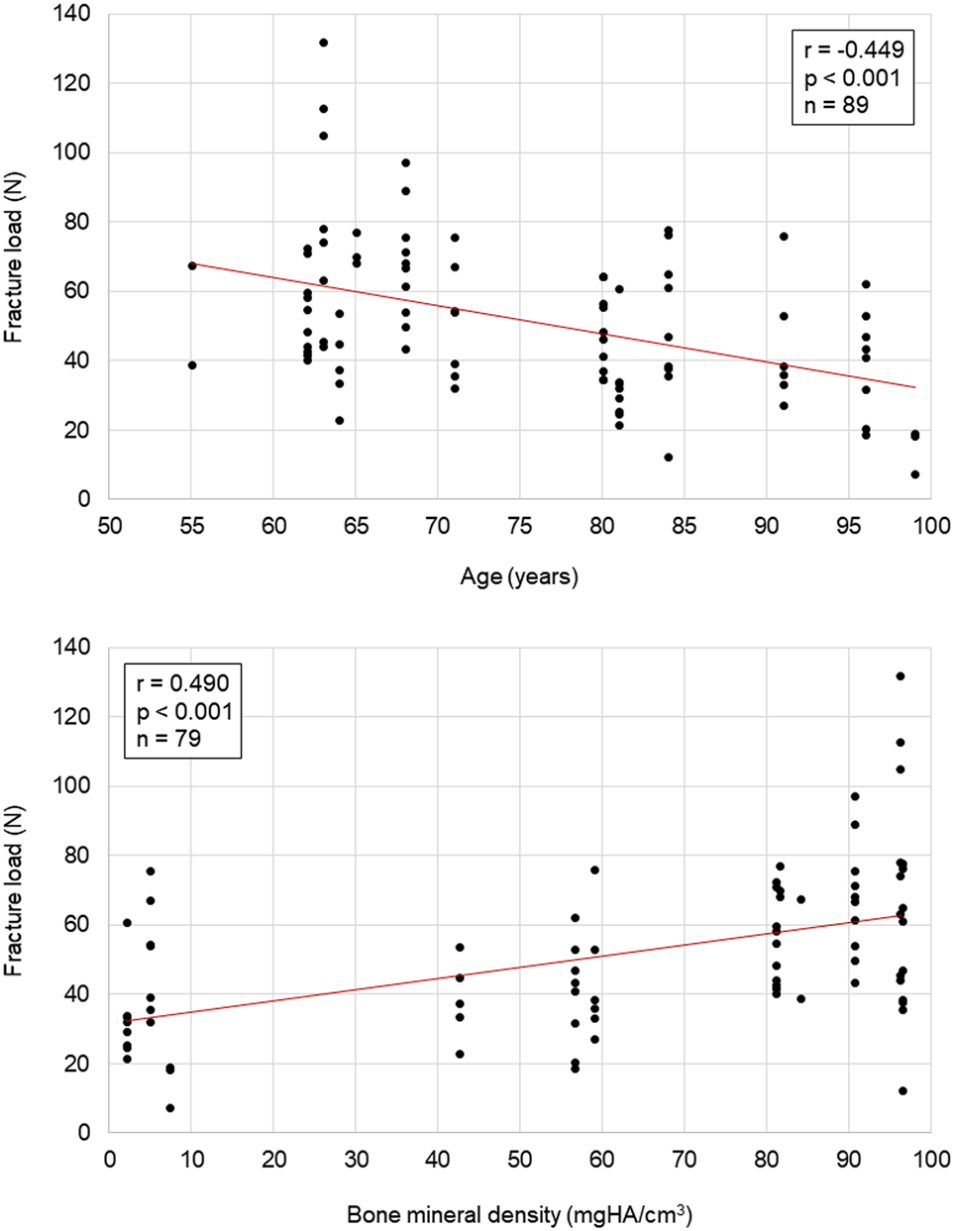
Scatter diagrams illustrating the effects of age (top) and bone mineral density (bottom) on fracture load. Trend lines are depicted in red.

Both age and bone mineral density showed medium correlations with fracture load and absorbed energy, while sex did neither significantly affect fracture load (*p* = 0.445) nor the other mechanical variables maximum displacement (*p* = 0.929), rib stiffness (*p* = 0.981), absorbed energy (*p* = 0.981), and maximum external and internal rib surface strains (*p* = 0.260/0.340). Age exhibited negative correlation coefficients with fracture load (r =  − 0.449, *p* < 0.001) and absorbed energy (r =  − 0.349, *p* = 0.001), meaning that the fracture load tended to decrease with increasing donor age, whereas bone mineral density showed positive correlation coefficients with fracture load (r = 0.490, *p* < 0.001) and absorbed energy (r = 0.382, *p* = 0.001), entailing a tendency towards increasing fracture load with increasing bone mineral density (Fig. 1).

### Effects of rib level and side

**Figure 2:**
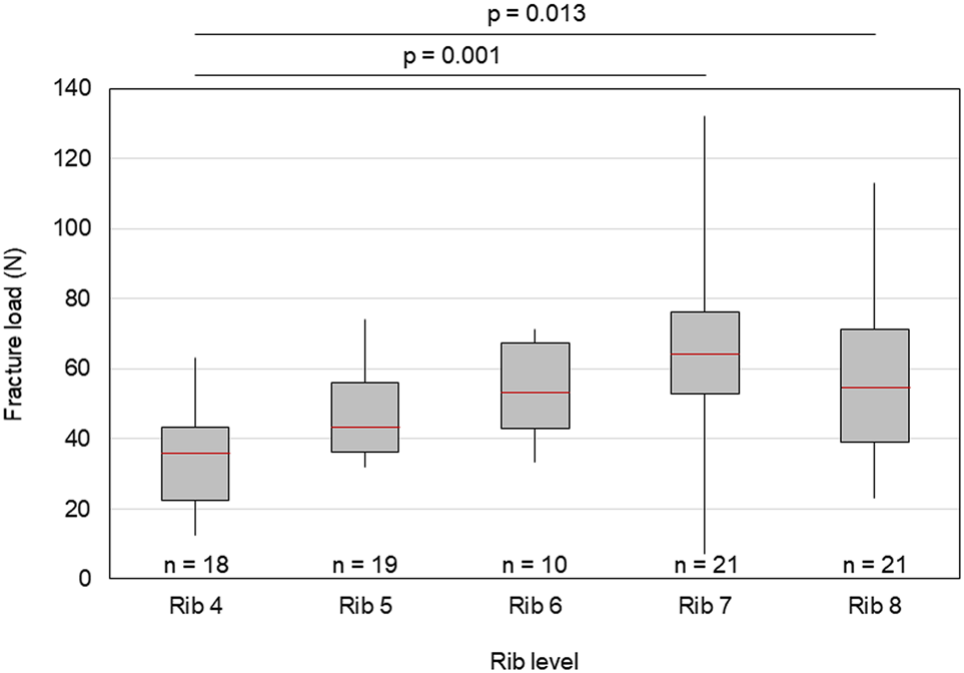
Boxplot diagram illustrating the effect of rib level on fracture load. Median values are depicted as red lines, the boxes include the 25th to 75th percentile, and the whiskers represent the maximum and minimum values.

Both fracture load and stiffness tended to increase gradually from rib 4 to rib 7, while slightly decreasing for rib 8 compared to rib 7. Fracture load and stiffness were significantly higher for rib 7 and rib 8 compared to rib 4 (fracture load: *p* = 0.001, *p* = 0.013; stiffness: *p* = 0.002, *p* = 0.007), respectively (Fig. 2). The rib cage side did neither significantly affect fracture load (*p* = 0.989) nor the other mechanical variables maximum displacement (*p* = 0.166), rib stiffness (*p* = 0.773), absorbed energy (*p* = 0.354), and maximum external and internal rib surface strains (*p* = 0.490/0.338).

### Effect of global rib morphology

Antero-posterior rib length exhibitedlow positive correlation with fracture load (r = 0.287, *p* = 0.006), whereas rib width, rib length/width ratio, and external rib edge length were not significantly correlated with fracture load. Medium correlation coefficients were found for stiffness with both rib width (r =  − 0.446, *p* < 0.001) and rib length/width ratio (r = 0.409, *p* < 0.001), while external rib edge length presented solely low correlation coefficients with maximum displacement (r = 0.248, *p* = 0.020).

### Effect of rib cross-sectional morphology

**Figure 3:**
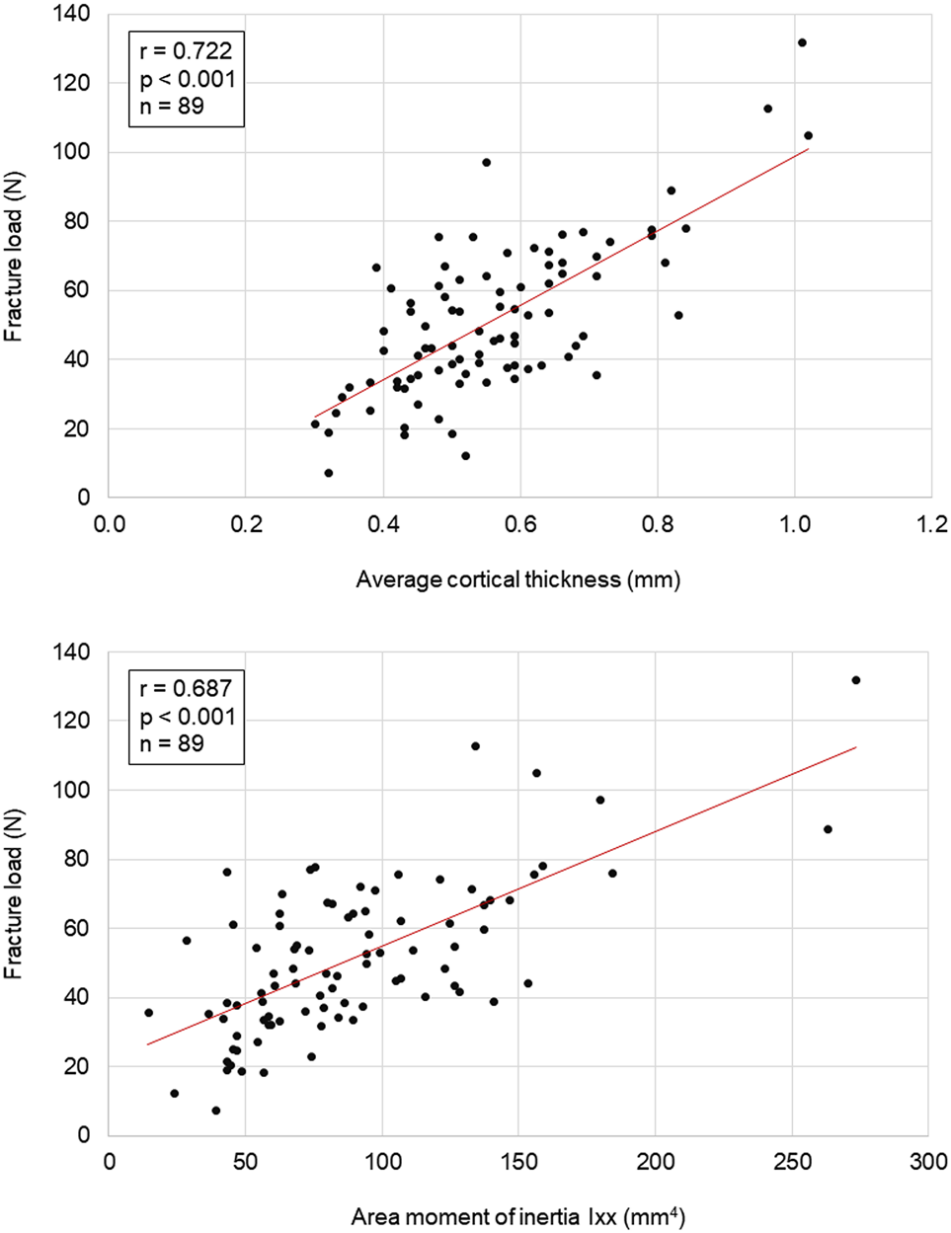
Scatter diagrams illustrating the effects of cortical thickness and area moment of inertia around the longitudinal axis of the rib cross-sectional area (xx) on fracture load. Trend lines are depicted in red.

Rib cross-sectional variables overall showed higher correlation coefficients with the mechanical variables compared to global rib morphological variables. Cortical thickness (r = 0.722, *p* < 0.001) and area moment of inertia around the longitudinal rib cross-sectional axis (r = 0.687, *p* < 0.001) exhibited high positive correlation with fracture load (Fig. 3). These two variables further showed high correlation coefficients with absorbed energy (cortical thickness: r = 0.617, *p* < 0.001; area moment of inertia around the longitudinal rib cross-sectional axis: r = 0.640, *p* < 0.001). Medium correlation coefficients with fracture load were found for bone volume/tissue volume ratio (r = 0.448, *p* < 0.001), trabecular number (r = 0.479, *p* < 0.001), and trabecular separation (r =  − 0.367, *p* < 0.001). The area moment of inertia around the transverse rib cross-sectional axis (r = 0.227, *p* = 0.033) exhibited a low correlation coefficient, while trabecular thickness (r = 0.201, *p* = 0.058) did not exhibit substantial correlation. High correlation coefficients were also detected for both bone volume/tissue volume ratio and trabecular number together with absorbed energy (r = 0.542, *p* < 0.001; r = 0.539, *p* < 0.001), maximum external rib surface strain (r = 0.631, *p* < 0.001; r = 0.648, *p* < 0.001), and minimum internal rib surface strain (r =  − 0.644, *p* < 0.001; r =  − 0.559, *p* = 0.001).

All results including the experimental raw data and the statistical variables are retrievable from the supplementary material files attached to the online version of this publication.

## Discussion

Rib fractures in elderly people represent a major clinical issue due to both increased risk of falling and higher mortality compared to the younger population, necessitating the exploration of mechanisms and promoting factors for rib fracture emergence. This study aimed to explore determinants of rib fragility using an experimental test design.

The outcomes of the present study indicated a combined, however hierarchically organized effect of the investigated continuous variables on human rib fragility. Based on the correlation analysis, solely considering statistically significant findings and arranging them in descending order according to their correlation coefficients, the following ranking order can be derived for increased rib fragility, defined as reduction in fracture load, which was considered as the primary indicator: (1) Low cortical thickness, (2) low area moment of inertia around the longitudinal rib cross-sectional axis, (3) low bone mineral density, (4) low trabecular number of the rib cross-section, (5) high age, (6) low bone volume/tissue volume ratio, (7) high trabecular separation of the rib cross-section, (8) shorter antero-posterior rib length, and (9) lower area moment of inertia around the transverse rib cross-sectional axis. Rib width, rib length/width ratio, external rib edge length, and trabecular thickness of the rib cross-section, in contrast, were not found to substantially affect rib fragility when defined as reduction in fracture load. Overall, correlation coefficients were relatively low, potentially indicating mutual dependency between single variables. Therefore, it can be assumed that there is no definite determinant of rib fragility, but a combination of several, differently weighted factors. Out of the discrete variables, rib level was found to be an important parameter that significantly affected rib fragility, in contrast to sex and rib cage side. While highest fracture resistance was found for rib levels 7 and 8, it gradually decreased in superior direction up to level 4. This might be explained by the effects of cortical thickness, which was significantly higher in rib 8 compared to level 4 (*p* = 0.022), and area moment of inertia around the longitudinal rib cross-sectional axis, which was significantly higher in levels 6 and 7 compared to level 4 (*p* = 0.015/0.036). Indirectly, rib level therefore might be a further essential determinant for rib fragility together with the above stated continuous variables.

Regarding clinically determinable parameters, rib level, bone mineral density, and age might be the most crucial determinants when assessing rib fragility of elderly patients, whereas sex and rib cage side might be negligible in clinical practice. While age and bone mineral density might be closely related to each other as well as to most of the analyzed morphological factors, rib level represents a remarkable determinant for rib fragility, which should be further investigated in future studies to explore its role regarding fracture resistance of the entire rib cage complex. Bone mineral density, on the other hand, represents a variable, which is affected by several influential factors due to its systemic character, such as diseases or nutritional and hormonal effects. The fact that sex was not affecting rib fragility in the present study, however, might indicate that bone mineral density can be seen as an independent determinant for rib fragility. In the present study, bone mineral density was evaluated from vertebral bodies, since there was no established procedure for ribs, which was seen as justifiable due to the systemic feature of bone mineral density^20^.

Rib fragility was predominantly affected by local, cross-sectional morphological factors rather than by global morphology in this study. Indeed, previous studies showed that both cortical thickness and cross-sectional area moment of inertia were significantly higher in lateral and posterior rib sections compared to the anterior rib section and increased with rib level^21,22^, corresponding to the findings of the present study, where fractures occurred primarily in the anterior and antero-lateral rib sections and fragility gradually decreased from rib levels 4–7. While the determinants affecting the cross-sectional rib shape are not known, it can be assumed that rib cross-sectional geometry is not optimally adapted to high antero-posterior rib cage compression as in case of frontal impacts, since the inner cortical bone is about 40% thicker compared to the outer cortical bone^22^. Antero-posterior rib length exhibited low positive correlation with fracture load, stiffness, and absorbed energy. Since the antero-posterior rib length was significantly increased at rib levels 6–8 compared to level 4 (*p* = 0.002/ < 0.001/ < 0.001) as well as at level 7 compared to level 5 (*p* = 0.002), corresponding to previous findings that the antero-posterior rib length approximately doubled from level 1 to level 7 and then slightly decreased^23^, increased antero-posterior rib length may therefore most probably reduce rib fragility indirectly via the rib level, which itself might be influenced by local, cross-sectional morphological determinants, such as the cortical thickness or the area moment of inertia.

In a previous in vitro study, investigating the fracture mode after antero-posterior compression in eight ribs, Daegling et al. detected five transverse, two oblique, and one multifragmentary fracture types^24^, corresponding to the findings of the present study, where of the 89 tested ribs, 60 exhibited transverse, 25 oblique, and four multifragmentary fracture types. However, the study of Daegling et al. solely showed four incomplete fractures in the eight tested ribs, whereas in the present study, 74 of the 89 fracture types were infractions, which might be explained by differences in testing conditions and the use of rehydrated rib specimens, since it was shown that moisture level can affect the fragility of cortical bone^25^. Compared to data of a retrospective clinical study, however, rib fracture locations of the present study showed similar patterns as ribs of cardiopulmonary resuscitated patients^19^, which might be explained by equivalent loading conditions in both cases. Therefore, it can be assumed that pure antero-posterior rib cage compression predominantly results in anterior or antero-lateral rib fractures. As in the present study, another large experimental study by Agnew et al.^13^ also detected bone mineral density, global rib geometry, and cross-sectional geometry as predictors of the structural properties of human ribs. However, cortical thickness did not appear to be a useful predictor in this study, which is in contrast to the findings of the present study and the numerical study of Li et al.^14^, whereas increasing age as a factor for increased rib fragility was also detected in most previous studies^9,11,13^. In contrast, sex exhibited major effects on the structural properties of human ribs in other studies^11,13^, which, however, could not be determined in the present study.

The present study entailed several limitations due to its experimental design. First, the boundary conditions of the test setup were simplified and did not exactly replicate the loading conditions during blunt chest trauma in terms of loading direction and rate. For instance, it was not feasible to align the rib ends reproducibly in antero-posterior loading direction without creating primary constraint forces due to the complex three-dimensional morphology of the individual ribs. Beyond that, a low loading rate was chosen in order to enable surface strain measurement, which requires low deformation velocities. Moreover, potential damping effects of the costal cartilage were neglected in order to ensure more reproducible testing conditions. Apart from that, the anterior PMMA embedding could have accelerated the fracture process by creating stress concentrations at the transition between rib and PMMA, which could not be prevented due to the experimental nature of this study. Furthermore, measurement inaccuracies of the load cell, the digital image correlation system, and the micro-CT might have affected the outcome variables and should be considered when interpreting the results of the present study. While inaccuracies are overall estimated low with regard to the accuracy ranges in case of the load cell and the digital image correlation system, potential inaccuracy in case of the micro-CT is also dependent from the evaluator and therefore difficult to estimate despite standardized evaluation techniques. Moreover, samples for micro-CT analysis were taken from the fracture site instead of a defined anatomical landmark in order to compare the morphological values of the weakest points of the respective ribs. While these were in the anterolateral part of the ribs in almost all cases, this might have affected the comparability of the morphological values nonetheless. One substantial limitation is the usage of fine sandpaper for the preparation of the rib surface in order to assure valid results with regard to surface strain measurements. However, the sandpaper was carefully used to remove remaining tendon tissue and the periosteum, which were assumed to have no distinct effect on the mechanical stability of the ribs, while care was taken not to remove bone tissue. Therefore, effects of this approach on the results were considered as low by the authors, but have, nevertheless, to be taken into account. Finally, regarding the statistical evaluation, the inclusion of multiple ribs from same donors also represents a limitation of this study.

In conclusion, reduced cortical thickness, area moment of inertia around the longitudinal axis of the rib cross-section, and bone mineral density predominantly promote rib fragility, whereas sex, global morphology, and rib cage side do not represent essential fragility determinants. When examining elderly patients for rib fractures due to blunt chest trauma, for instance following falls from low height, especially patients with low bone mineral density and the mid-thoracic area of the patients should therefore be carefully checked.

## Methods

### Specimens

**Figure 4:**
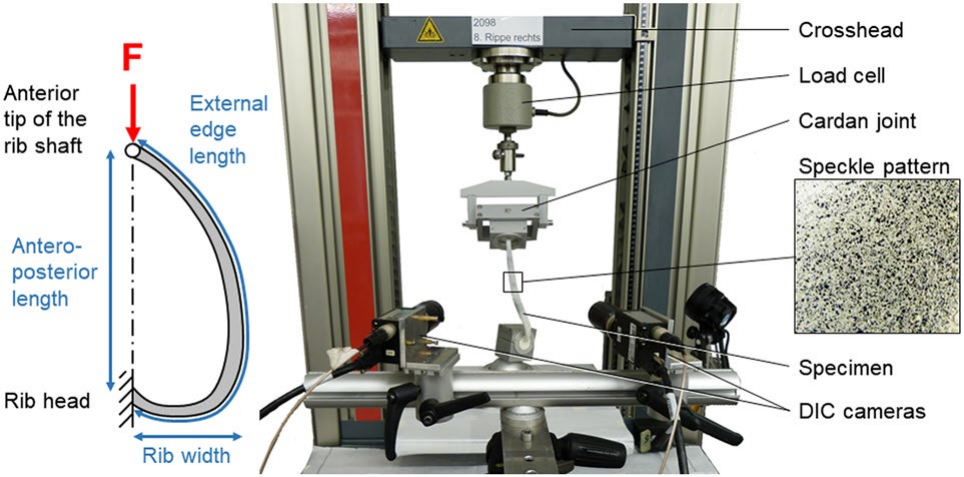
Illustration of the test setup.

A total of 89 human ribs from 13 donors were prepared for experimental testing, while solely ribs from levels 4 to 8 were included since highest fracture rates were detected at these levels in a recent retrospective study^19^. Donor age ranged from 55 to 99 years with a mean of 75 years, including seven male and six female donors and a total of 45 left and 44 right ribs (Table 1). The use of the specimens was approved by the ethics committee board of the University of Ulm (vote no. 92/20). The specimens were acquired from body donation programs which require legally valid informed consents from a parent or legal guardian (Science Care Inc., Phoenix, USA; Anatomy Gifts Registry program, Hanover, USA). All methods were performed in accordance with relevant guidelines and regulations and following the Declaration of Helsinki. Rib cage specimens were gathered fresh frozen and stored at − 20 °C. Prior to preparation, bone quality was assessed by CT scans (Somatom Definition AS, Siemens Healthcare, Erlangen, Germany) with a resolution of 512 × 512x804 pixels and a voxel size of 0.87 × 0.87x0.6 mm in terms of bone mineral density of the respective spines. The bone mineral density values were evaluated from the trabecular bones of the lumbar vertebral bodies L2, L3, and L4 using standard clinical density-reference phantoms (Osteo Phantom, Siemens Healthineers, Erlangen, Germany), which were included in the patient table of the CT device and averaged for every specimen, while the calibration curves were automatically fitted using a standardized software protocol for clinical evaluation, comparable to the procedure of a previous investigation^26^. After thawing the specimens at 5 °C for about 12 h, the ribs were separated from the spines by cutting the connecting ligaments. All muscular and ligamentous tissues were carefully removed and the costal cartilage was severed close to the cartilage-bone junction. Ribs showing any signs of bony defects, previous fractures, or degeneration were excluded from the study. Rib heads and shafts were coaxially embedded in a polymethylmethacrylate cylinder (Technovit 3040, Heraeus Kulzer, Wehrheim, Germany) for a length of about 40 mm, while care was taken that the rib was uniformly surrounded by PMMA to ensure rigid fixation in the testing device. After preparation, the ribs were frozen again and stored at − 20 °C in sealed polyethylene bags in order to keep the exposure to room temperature to the same time period for all ribs. Prior to testing, every rib was thawed for about 12 h in 0.9% saline solution. Immediately before testing, remaining tendon tissue and the periosteum were carefully removed using fine sandpaper while taking care not to damage bone substance. In the next step, the ribs were primed with a thin layer of white water-based paint and sprayed with a black speckle pattern for surface strain measurement with digital image correlation (Fig. 4). This speckle technique was verified in a preliminary study not to affect the biomechanical properties of biological tissue^27^.

**Table 1:** Data on specimens used for this in vitro study.

Donor ID	Sex	Age in years	Rib level and side (l = left / r = right)
1	Female	55	8 l/r
2	Male	62	4 l/r, 5 l/r, 6 l/r, 7 l/r, 8 l/r
3	Male	63	4 l/r, 5 l/r, 7 l/r, 8 l/r
4	Female	64	6 l, 7 l/r, 8 l/r
5	Female	65	5 l, 6 l, 8 l
6	Male	68	4 l/r, 5 l/r, 6 l/r, 7 l/r, 8 l/r
7	Female	71	4 l, 5 l/r, 7 l/r, 8 l/r
8	Male	80	4 l/r, 5 l/r, 6 l/r, 7 l/r, 8 l/r
9	Male	81	4 l/r, 5 l/r, 7 l/r, 8 l/r
10	Female	84	4 l/r, 5 l/r, 6 l/r, 7 l/r, 8 r
11	Male	91	4 l/r, 5 l/r, 7 r, 8 r
12	Male	96	4 l/r, 5 l/r, 7 l/r, 8 l/r
13	Female	99	4 r, 7 l/r

### Biomechanical testing

**Figure 5:**
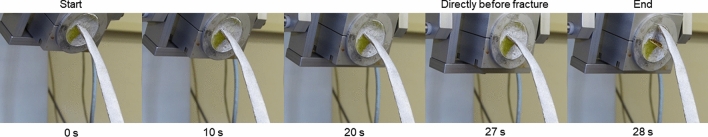
Image sequence of a typical loading test.

The specimens were loaded in antero-posterior compression until fracture using a material testing machine (Zwick Roell Z010, Zwick Roell AG, Ulm, Germany) equipped with a 200 N load cell (Hottinger Baldwin Messtechnik GmbH, Darmstadt, Germany) (Fig. 4), having a measuring error of < 0.3%. Load and displacement data was acquired using the software testXpert II (Zwick Roell AG, Ulm, Germany) with a sampling frequency of 100 Hz. To create loading conditions that were as reproducible as possible, the posterior end of the rib was rigidly fixed in a ball joint, which was adjusted in a way that the rib head and the anterior end of the rib shaft were preferably aligned in loading direction without creating constraint forces on the rib, while the anterior rib end was clamped in a cardan joint allowing quasi-physiological rotations. Loading was performed in antero-posterior direction with a constant velocity of 1 mm/s after applying a preload of 5 N to remove any effect of mechanical play between the components of the loading system (Fig. 5). Fracture occurrence was determined from the decline in the load–displacement curve after combined audible cracking of the bone and appearance of a clearly visible fracture line. Simultaneously with load application, full-field analysis was performed using a 3D digital image correlation system (Q400, Dantec Dynamics, Denmark) consisting of two cameras in order to measure the surface strain (Fig. 4), exhibiting a relative displacement error of < 1/3 pixels and a strain error of < 180 μStrain. This technique has been validated and successfully applied to the spine in previous in vitro studies^28–31^. To detect maximum and minimum surface strains during rib deformation, about half of the ribs were examined from a lateral view and the other half from a medial view, while care was taken to evenly distribute the specimens with respect to age, sex, bone mineral density, rib cage side, and rib level.

### Morphological analysis

**Figure 6:**
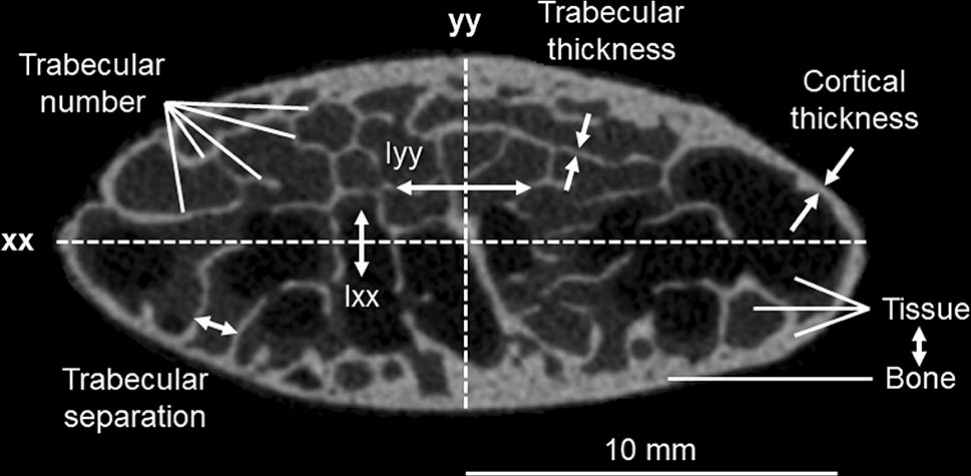
Illustrative summary of all evaluated cross-sectional morphological parameters.

After each trial, fracture type and location were visually assessed and documented. Cross-sectional rib morphology was acquired using micro-CT scans (Skyscan 1172 Micro-CT, Skyscan, Kontich, Belgium) of about 10 mm long samples, which were cut posteriorly from the fracture position and as closely as possible to the fracture site, respectively. The scans were performed using a tube voltage of 100 kV, a tube current of 100 µA, an Al-Cu filter for beam hardening reduction, and a scan period of 38 min, obtaining an isotropic voxel size of 5 µm. The following parameters were examined using a standard threshold technique for greyscale values within the software CTAn 1.17.7.2 (Skyscan, Kontich, Belgium) in combination with a calibration phantom (Bruker MicroCT, Kontich, Belgium): Bone volume/tissue volume ratio, average thickness, number, and separation of the trabeculae, average cortical thickness, and area moments of inertia Ixx and Iyy around the longitudinal (xx) and transverse (yy) cross-sectional axes (Fig. 6). All values were automatically calculated by the software for every cross-section and finally averaged for a determined region of interest over 50 adjacent sample slices. Thickness values were defined as the minimum thickness at different locations of the respective area. Ixx and Iyy were defined as the integral of the quadratic distance of all bony elements to the axes xx and yy over the cross-sectional bony area, reflecting potential resistance against bending deformation around these axes. Global morphology of the ribs was identified regarding rib length and width prior to embedding using a tape measure. Rib length was measured as both the external edge length, characterizing the length of the external rib side from rib head to anterior tip of the rib shaft, and the antero-posterior length, describing the direct distance between rib head and anterior tip of the rib shaft (Fig. 4). Rib width was defined as the maximum distance between the rib shaft and the connecting line between rib head and anterior tip of the rib shaft.

### Data processing and statistics

Load–displacement data were post-processed using Matlab 2018 (MathWorks Inc., Natick, USA) in order to calculate fracture load, crosshead displacement at fracture, apparent stiffness and absorbed energy, while the displacement was first set manually to zero for the starting point of load increase. Stiffness was defined as the gradient of the load–displacement curve within the interval starting from 5 N and ending at the point where 20% of the peak load were reached, while absorbed energy was specified as the area under the load–displacement curve up to fracture. All biomechanical, morphological, and donor-specific data were collected and post-processed using Excel 2016 (Microsoft Corp., Redmond, USA). Statistical analyses were performed in SPSS 24 (IBM Corp., Armonk, USA). For all continuous variables, linear relationships of continuous variables were investigated using the Pearson correlation coefficient r defining |r|≤ 0.1 as no linear correlation, 0.1 <|r|≤ 0.3 as low linear correlation, 0.3 <|r|≤ 0.5 as medium linear correlation, and |r|> 0.5 as high linear correlation according to the recommendation of Cohen^32^. For all statistical analyses, the significance level was set to 0.05. For comparisons between two groups, the Mann–Whitney U test was used, whereas for comparisons between multiple groups, a Kruskal–Wallis test with Dunn-Bonferroni post hoc correction was performed. Information on which variables were treated as independent and which as dependent is given in Table 3 and Table 4 of the supplementary material file.

### Approval for human experiments

The use of the specimens was approved by the ethics committee board of the University of Ulm (92/20).

## Supplementary Information

Supplementary Legends.

Supplementary Information 1.

## Data Availability

All data including the experimental raw data and the statistical variables are retrievable from the supplementary material file attached to the online version of this publication.
