# Supplementary material for: Experimental study exploring the factors that promote rib fragility in the elderly
Source: Sci Rep. 2021 Apr 29;11:9307. doi: 10.1038/s41598-021-88800-9 (PMC8085244; doi:10.1038/s41598-021-88800-9)
Supplement: Supplementary file 1 — Supplementary Legends. [file 41598_2021_88800_MOESM1_ESM.docx]

**Table 2.** Supplementary material. Raw data of the experiments.

**Table 3.** Supplementary material. Statistics: Pairwise comparisons.

**Table 4.** Supplementary material. Statistics: Correlation coefficients.
